# Enhanced Stretchable and Sensitive Strain Sensor via Controlled Strain Distribution

**DOI:** 10.3390/nano10020218

**Published:** 2020-01-27

**Authors:** Huamin Chen, Longfeng Lv, Jiushuang Zhang, Shaochun Zhang, Pengjun Xu, Chuanchuan Li, Zhicheng Zhang, Yuliang Li, Yun Xu, Jun Wang

**Affiliations:** 1 Fujian Provincial Key Laboratory of Functional Marine Sensing Materials, Center for Advanced Marine Materials and Smart Sensors, Minjiang University, Fuzhou 350108, China; zhicheng@mju.edu.cn (Z.Z.);; 2 Institute of Semiconductors, Chinese Academy of Sciences, Beijing 100083, China; lflv@semi.ac.cn (L.L.); jszhang@semi.ac.cn (J.Z.); sczhang@semi.ac.cn (S.Z.); lichuan@semi.ac.cn (C.L.); 3 Faculty of Clothing and Design, Minjiang University, Fuzhou 350108, China; xupj@mju.edu.cn

**Keywords:** stretchable, strain sensor, graphene, sensitive, strain distribution

## Abstract

Stretchable and wearable opto-electronics have attracted worldwide attention due to their broad prospects in health monitoring and epidermal applications. Resistive strain sensors, as one of the most typical and important device, have been the subject of great improvements in sensitivity and stretchability. Nevertheless, it is hard to take both sensitivity and stretchability into consideration for practical applications. Herein, we demonstrated a simple strategy to construct a highly sensitive and stretchable graphene-based strain sensor. According to the strain distribution in the simulation result, highly sensitive planar graphene and highly stretchable crumpled graphene (CG) were rationally connected to effectively modulate the sensitivity and stretchability of the device. For the stretching mode, the device showed a gauge factor (GF) of 20.1 with 105% tensile strain. The sensitivity of the device was relatively high in this large working range, and the device could endure a maximum tensile strain of 135% with a GF of 337.8. In addition, in the bending mode, the device could work in outward and inward modes. This work introduced a novel and simple method with which to effectively monitor sensitivity and stretchability at the same time. More importantly, the method could be applied to other material categories to further improve the performance.

## 1. Introduction

Stretchable and wearable electronics have attracted tremendous interest due to their broad prospects in health monitoring and epidermal applications. Various stretchable devices such as displays [1,2], integrated circuits [3,4], energy conversion and storage units [5,6,7,8], and sensing systems [9,10] have been developed to improve practicality. As one of the most important and typical devices, strain sensors have been the subject of remarkable progress in terms of sensing mechanisms including strain–resistance effect [11,12,13,14,15,16,17], strain–capacitance effect [18,19,20,21,22,23], thermoelectric effect [24,25], piezoelectric effect [26,27,28,29,30], triboelectric effect [31,32,33,34,35], and field effect [36,37,38]. There are also other methods for strain sensing based on polymer optical fibers [39,40,41]. Resistive strain sensors stand out from these other types of device due to their excellent characteristics, including abundant material source, simple fabrication process, high gauge factor, and easy integration. Many efforts have been made to improve their sensitivity and stretchability for practical applications. To date, the sensitivity of the resistive strain sensor has reached over 5000 [42,43,44], and the stretchability has exceeded 900% [45,46,47].

Various materials including organic materials [48,49], carbon materials [50,51], metal materials [52,53], semiconductor materials [54,55], and hybrid materials [56,57] have been utilized to fabricate resistive strain sensors with better performance. Diverse novel materials have been introduced to enhance device sensitivity. Instead of traditional materials, nanowires or nanofibers are usually adopted [11,17]. Cracks can also be introduced into the material to effectively increase the sensitivity [13,44]. Alongside sensitivity, stretchability is equally important. Microstructures such as crumple structures, zigzag structures, U-shape structures, and mesh layout are usually introduced into the conductor surface to form highly stretchable strain sensors [58,59]. In addition, intrinsic stretchable materials such as ionic conductive hydrogel can be selected to improve device stretchability [45,47,60]. Nevertheless, it is hard to take both sensitivity and stretchability into consideration at the same time. Ultrasensitive and highly stretchable resistive strain sensors are still a great challenge.

In this work, we demonstrated a straightforward method by which to fabricate a highly sensitive and stretchable graphene-based strain sensor. By connection of planar graphene and crumpled graphene (CG) films with various crumple degrees, we were able to effectively modulate the sensitivity and stretchability of the device. For stretching mode, the device was made up of two CG nanosheets with planar graphene between them. This device showed a gauge factor (GF) of 20.1 with 105% tensile strain, and it could endure a maximum tensile strain of 135% with a GF of 337.8. In addition, for bending mode, the device consisted of two planar graphene films with a CG film between them. The device could work in outward and inward modes. This work introduced a novel and simple strategy by which to effectively monitor sensitivity and stretchability at the same time. More importantly, the strategy could be applied to other material categories.

## 2. Materials and Methods

### 2.1. Fabrication of the Highly Stretchable and Sensitive Strain Sensor

**Figure 1:**
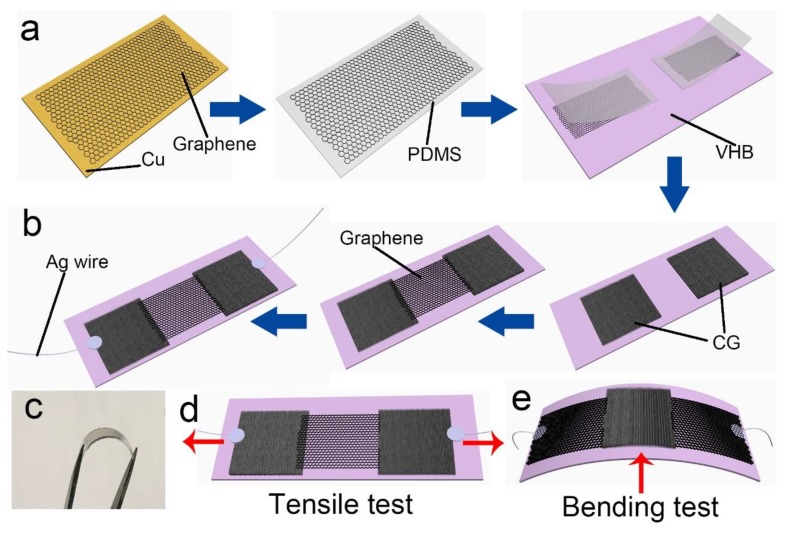
Schematic diagram of the fabrication process of the highly sensitive and stretchable strain sensor. (**a**) The transfer printing of graphene. (**b**) The fabrication process of a strain sensor. (**c**) Photograph of the flexible device. (**d**) Schematic diagram of the strain sensor under tensile testing. (**e**) Schematic diagram of the strain sensor under bending testing.

Graphene grown on a copper substrate was transferred onto a polydimethylsiloxane (PDMS) thin film by transfer printing method. Two graphene/PDMS films were then attached to a uniaxially pre-stretched very-high-bond (VHB) film. The graphene was left on the pre-stretched VHB after peeling the PDMS film, as shown in Figure 1a. After releasing the pre-strain, two CG sheets formed the VHB film, leaving a gap in the middle. Later, a planar graphene film was transferred onto the VHB film which was located in the middle of the two CG regions. The planar graphene sheet covered the blank area. Finally, a stretchable and sensitive strain sensor was achieved after leading wire and packaging, as illustrated in Figure 1b. A photograph of the highly flexible device is shown in Figure 1c. This device structure was mainly designed for tensile applications. In addition, bending situations are normal in joint movement. A structure of planar graphene/CG/planar graphene was then improved for bending applications. Schematic illustrations of the tensile and bending tests are shown in Figure 1d,e.

### 2.2. Characterization of the Device

The surface structure of the materials was characterized using a scanning electron microscope (SEM, Quanta 450, FEI Company, Hillsborough, CA, USA), and the resistance was measured using a multimeter (DMM 7510, Keithley, Cleveland, OH, USA). The stability test and the current–voltage curve of the device were assessed using a semiconductor characterization system (B1500A, Agilent, Santa Clara, CA, USA) with commercial slides.

## 3. Results and Discussion

**Figure 2:**
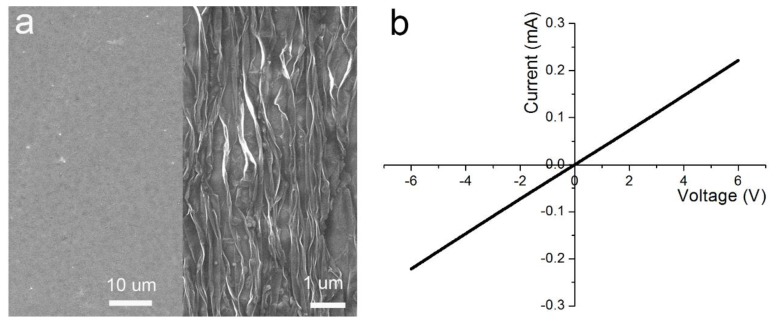
The interface characteristics of the graphene/crumpled graphene (CG). (**a**) The SEM pictures of the graphene and CG. (**b**) I–V curve of the graphene/CG junction.

First, the surface morphology of the device was analyzed, as shown in Figure 2. Figure 2a shows the SEM picture of graphene (left) and CG (right) on the substrate. Under a uniaxially pre-strained situation, parallel ridges were discovered on the surface. The crumples and buckles supported the stretchability, as shown on the right side of Figure 2a. During fabrication of the strain sensor, the graphene was directly attached onto the CG. The CG on the VHB film affected the transfer printing quality of the graphene because it made the VHB less sticky. Work function differences between the graphene and CG were possible, so it was necessary to analyze the homojunction of graphene/CG. The contact resistance test was performed using the Agilent B1500A and the results are exhibited in Figure 2b. From the I–V curve of graphene/CG, the homojunction was ohmic contact, and the contact resistance was about 3 kΩ. The ohmic contact of graphene/CG offers benefits not only for the strain sensor, but also for other electronic applications.

**Figure 3:**
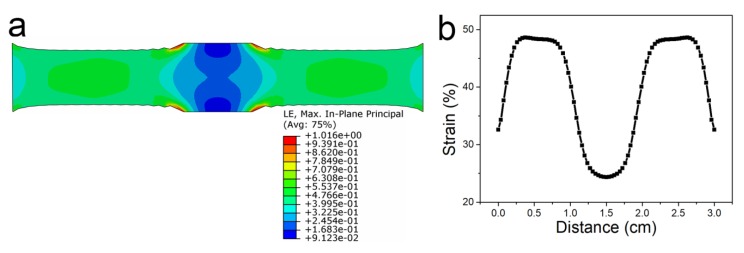
The mechanical simulation of the device. (**a**) The simulation of the strain sensor under uniaxial stretching. (**b**) The strain distribution of the strain sensor under simulated stretching.

To rationally design the parameters of the device, we simulated the strain distribution of the device under tensile strains, as shown in Figure 3. Figure 3a shows the strain distribution of the device under tensile strain. The size of the device was 2.0 cm in length and 0.5 cm in width. The two ends of the device (up and down) were fixed, and the other two ends were stretched. We ran a finite element analysis calculation to simulate the strain distribution. The Young’s modulus and the Poisson’s ratio in this simulation were 1 × 10^6^ Pa and 0.49, respectively. It was clearly observed that the strain in both ends of the device was relatively larger, while it was very small in the middle area. The simulation result shown in Figure 3a was correlated with our previous work [51]. The detailed strain distribution is displayed in Figure 3b. Under a tensile strain of 50%, the strain in the middle position was only about 24%. According to the strain distribution in the simulation, we designed the device structure by combining two types of strain sensor. One was the highly stretchable strain sensor with a small GF, which was located on the two ends of the VHB. The other was the highly sensitive strain sensor with a narrow working range. To extend the working range of the strain sensor without sacrificing its sensitivity, planar graphene and CG were combined in a rationally designed structure.

**Figure 4:**
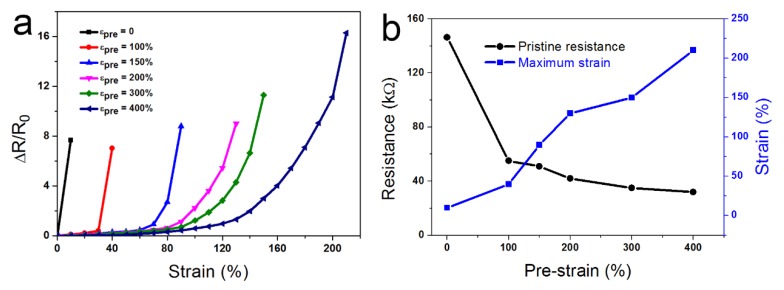
The electromechanical properties of various strain sensors. (**a**) The relationship between the resistance variation and strain with various pre-strains. (**b**) The resistance and the maximum endurable strain comparison of various strain sensors with different pre-strains.

The electromechanical properties of the strain sensors with various crumple degree are characterized in Figure 4. The crumple degree was defined as $\varepsilon_{\mathrm{pre}}\;=\;\left(L_{S}\;-\;L_{P}\right)/L_{P}$ where $L_{S}$ and $L_{P}$ are the pre-stretched length and pristine length of VHB. The strain sensors with different $\varepsilon_{\mathrm{pre}}$ values showed significant differences in resistance–strain relationships, as shown in Figure 4a. The strain sensor based on planar graphene exhibited high sensitivity but poor stretchability. Under 10% tensile strain, cracks appeared on the graphene, which greatly increased its resistance. The sensitivity was characterized by the gauge factor (GF), which is defined as

(1) $$\mathrm{GF}\;=\;\frac{\Delta R/R_{0}}{\varepsilon }$$

where ΔR and $R_{0}$ are the resistance variation under tensile strain and the pristine resistance with no strain, respectively, and ε is the tensile strain applied to the sensor. The GF of all the strain sensors was calculated by linear fit.

Generally, the planar graphene was not able to endure 10% tensile strain. In this experiment, this was due to the wavy structure formed while transferring the planar graphene onto the VHB film. The strain sensor based on the CG showed better stretchability compared to the planar device. Furthermore, its working range under tensile strain was increased with increasing $\varepsilon_{\mathrm{pre}}$. The detailed characteristic of these sensors with various $\varepsilon_{\mathrm{pre}}$ was listed in Table 1. The strain sensors with $\varepsilon_{\mathrm{pre}}$ values from 100% to 400% exhibited a GF of about 1. Although the stretchability increased gradually, the sensitivity was low. Furthermore, the linear correlation (R^2^) decreased as the stretchability increased, meaning that the GF was further decreased in the same linear fit range. Errors may have been introduced in the linear fit. It should be noted that the limit of the VHB is about 500%, and our device approached this limit. The pristine resistance and the maximum tolerant strain of these strain sensors is displayed in Figure 4b. The pristine resistance of the planar graphene was about 150 kΩ and the resistance decreased rapidly as the $\varepsilon_{\mathrm{pre}}$ increased. The maximum tolerant strain is much larger than the linear fit range. Under the maximum strain, the resistance of the sensor exceeded the measurement range of the multimeter (100 MΩ).

**Figure 5:**
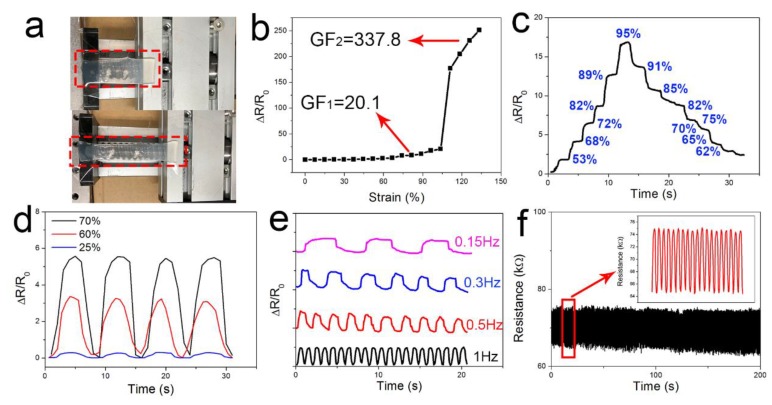
The characteristics of the strain sensor under stretching mode. (**a**) Experimental pictures of the device. (**b**) Resistance variation of the device under various tensile strain. (**c**) Resistance variation of the device under tensile strain and released conditions. (**d**) Response of the strain sensor under various strain conditions. (**e**) Response of the strain sensor under different frequency conditions. (**f**) Stability test of the device.

The properties of the designed strain sensor are characterized in Figure 5. The device was fixed to a commercial slide, as shown in Figure 5a. First, we measured the resistance variation of the device under various tensile strains. The relationship between the $\Delta R/R_{0}$ and ε is exhibited in Figure 5b. With small strains (ε < 105%), the resistance increased slowly due to the stretchability of the crumple structure. The GF obtained by the linear fit was 20.1. As we continued to increase the strain (ε ≥ 105%), cracks were formed. These cracks led to a rapid resistance increase. The sharp change of the resistance resulted in a high GF of 337.8. It should be noted that once small cracks had been formed, the device could not fully recover to its original state. Thus, the effective working range of the device was 105%. The key parameters are displayed in Table 2.

The typical response curve of the device is displayed in Figure 5c. The resistance needed enough time to return to its original state, so the curve is a little slanted. The device was first stretched to 95%, and then the strain was gradually released. In the stretching process, the resistance increased as the tensile strain increased from 53% to 95% and the resistance was stable. In the releasing process, the resistance started to recover. As it took time to return to its original state, the resistance exhibited as a slope. In addition, we performed multiple loading–unloading tests under different tensile strains of 25%, 60% and 70%, respectively. The results are shown in Figure 5d. The variation of the resistance under various frequencies is displayed in Figure 5e. The curves showed good repeatability. Finally, stability tests were performed, as shown in Figure 5f. From Figure 5, we can see that the sensitivity of the device was 20.1 in the working range of 105% and 337.8 in the range of from 105% to 135%. To date, the GF of the ultrasensitive strain sensor was about 40,000 [42] and the stretchability of the ultra-stretchable strain sensor reached up to 950% [47]. Although the sensitivity or the working range did not reach best possible level, it did reach a high level and show good potential in both areas at the same time. More important, it possessed the potential to modulate the sensitivity and stretchability of the device for various practical applications.

**Figure 6:**
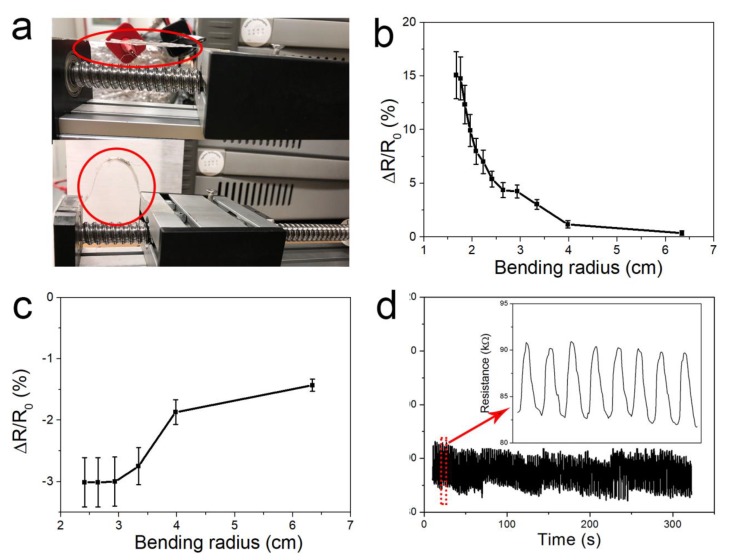
The characteristics of the device under bending condition. (**a**) Pictures of the test experiment. The resistance variation of the strain sensor under different bending radii of (**b**) outward bending and (**c**) inward bending. (**d**) Stability test of the device.

Furthermore, we designed another device for bending applications. The structure of the device was planar graphene/CG/planar graphene. The experimental results were shown in Figure 6. The device was attached onto a flexible thin film for the bending test. The bending radius of the device was controlled by a commercial slide, as shown in Figure 6a. The results of the bending test are exhibited in Figure 6b,c. In the outward bending test, the $\Delta R/R_{0}$ increased from 0.4% to 15.1% as the bending radius decreased from 6.3 cm to 1.7 cm. As the bending radius decreased from 2.4 cm to 1.7 cm, the resistance of the device increased rapidly. In the inward bending test, the $\Delta R/R_{0}$ got up to −3% as the bending radius deceased to 2.9 cm. The resistance did not continue decreasing as we further decreased the bending radius. The decreased resistance was due to the overlap of the crumpled graphene films. The newly formed path decreased the resistance. A stability test was conducted and the results are shown in Figure 6d. The resistance here was relatively higher than that in Figure 5f. This was due to the different structural design. The device contained more planar graphene, which has a higher resistance than that of CG. This device is suitable for wearable applications such as monitoring joint movement.

## 4. Conclusions

In summary, we demonstrated a straightforward method by which to construct a flexible strain sensor with high sensitivity and stretchability. According to the strain distribution in the simulation results, the strain was small in the middle area while it was large at both ends. The sensitivity and stretchability of the device could be effectively controlled by rational connection of planar graphene and CG. For stretching applications, the device was made up of two CG nanosheets with a planar graphene film between them. This device showed a GF of 20.1 at the working range of 105%, and it could bear a maximum tensile strain of 105% with a GF of 337.8. In addition, for the bending mode, the device consisted of two planar graphene films with CG between them, which revealed good stability. This work introduced a novel and simple strategy to effectively monitor sensitivity and stretchability at the same time. More importantly, the strategy could be applied to other material categories to further enhance the performance of strain sensors. The sensitive and stretchable sensor is promising for wearable applications and epidermal applications, especially in joint monitoring.

## Figures and Tables

**Table 1:** Comparison of various strain sensors with different pre-strains.

Pre-Strain Degree	Linear Fit Range	R^2^	GF
100%	0–30%	0.97474	1.31
150%	0–60%	0.96328	0.779
200%	0–80%	0.93992	0.753
300%	0–90%	0.91959	0.734
400%	0–110%	0.8364	0.641

**Table 2:** The key parameters of this strain sensor.

Linear Fit Range	R^2^	GF
0–105%	0.9138	20.1
105–135%	0.9764	337.8
